# Investigating the Role of Brain Lateralization and Gender in Paranormal Beliefs

**DOI:** 10.32598/BCN.9.10.923.1

**Published:** 2019-11-01

**Authors:** Abdolvahed Narmashiri, Ahmad Sohrabi, Javad Hatami, Azita Amirfakhraei, Shaniya Haghighat

**Affiliations:** 1. Institute for Cognitive Sciences Studies,ShahidBeheshti University, Tehran, Iran.; 2. Iranshahr University of Medical Sciences, Iranshahr, Iran.; 3. Department Psychology, Faculty of Humanities and Social Sciences, University of Kurdistan, Sanandaj, Iran.; 4. Department Psychology, University of Tehran, Tehran, Iran.; 5. Department of Psychology, Young Researchers and Elite Club, Islamic Azad University, Bandar Abbas Branch, Bandar Abbas, Iran.; 6. Department Psychology, University of Science and Culture, Tehran, Iran.

**Keywords:** Brain lateralization, Paranormal beliefs, Gender

## Abstract

**Introduction:**

Brain lateralization is associated with human behavior. Therefore, this study aimed at investigating the effects of brain lateralization on the scores of paranormal beliefs.

**Methods:**

The study population included 180 students of Sanandaj universities, Sanandaj City, Iran who were selected with convenience sampling method (100 left-brained males, 6 left-brained females, 56 both left- and right-brained males and 22 both left- and right-brained females). The research tools were the paranormal belief scale developed by Blackmore (1994), as well as the brain lateralization questionnaire (1985).

**Results:**

The obtained findings suggested a significant difference between the left-brain and right-brained people in terms of paranormal beliefs. A significant difference was also found between the left-brained males and both left- and right-brained females in terms of paranormal beliefs.

**Conclusion:**

The paranormal beliefs of the left-brained cases were different from both left- and right-brained subjects, which can be seen between the left-brained males and both left- and right-brained females.

## Highlights

- Paranormal beliefs change brain functional.
- Brain activity in paranormal believers differs from normal people.
- The brain functions of men and women differ in paranormal beliefs.

## Plain Language Summary

Cognitive and brain functions are important topics in paranormal beliefs. It seems that by understanding the brain and cognitive functions in paranormal beliefs, its implications can be better understood. An important point in paranormal beliefs is the tendency for women to be more than men. We sought to measure brain functions in paranormal beliefs and we evaluate the brain functions of men and women in paranormal beliefs.

## 1. Introduction

The human body seems to be symmetrical; however, many internal organs of the body are asymmetrical. The structure of the brain appears to be symmetrical, but the two hemispheres act differently, implying that they are asymmetrical (Tsujii, Okada & Watanabe, 2010). At first glance, the two hemispheres are reflections of each other; however, an accurate examination suggests their differences. Brain dissections reveal that the left hemisphere is always bigger than the right hemisphere. Additionally, there are substantial long nerve fibers in the right hemisphere, forming connections between many areas, which are far from the brain. In the left hemisphere, however, several short nerve fibers are facilitating many communications in one limited area (Atkinson et al., 2000).

Brain lateralization has different functions in human behavior (Lentz, 2018). It starts at birth and completes in 8 or 10 years (Davidson, 1994). It has been reported that the processing and organization of the cerebral cortex are different in the right- and left-handed people (Rodway, Wright, & Hardie, 2003). Advancements in electrophysiological studies have presented and supported lateralization and the fact that each hemisphere has its specific function. It is believed that both brain hemispheres equally contribute to cognitive functions (Molfese & Segalowitz, 1988). Those with lateralized brains have outstanding capabilities in comparison with others. In other words, a lack of lateralization is considered a developmental disorder (Beaton, 2019). Those with cognitive functions, which are less coordinated in the left hemisphere, are better in reasoning issues (either verbal reasoning or deductive reasoning). A less-known cognition pattern is observed in the left-handed people, both left- and right-handed people, and some right-handed people who have left-handed relatives (Hibard, 1997).

Paranormal beliefs have been defined as issues that violate the fundamental principles of science (Broad, 1949). Paranormal beliefs are hardly consistent with contemporary science findings and it is unlikely that the mental events directly affect a physical event (Broad, 1949). It is worth mentioning that paranormal beliefs lack universal accepted definitions (Wiseman & Watt, 2006). However, according to Gallup Poll, the paranormal beliefs are still widespread in societies despite much evidence against it (Mohr, Bracha & Brugger, 2003). Some studies have shown that gender can affect paranormal beliefs. Women, for example, show stronger belief in paranormal phenomena in comparison with men (Irwin, 1993; Rice, 2003). Besides, other studies have considered the effect of culture (Mohr et al., 2003). It should be noted that the perceptional processing and attention to paranormal beliefs are highly influenced by previous expectations, experiences, and learning. Paranormal beliefs are associated with perceptual orientations (Van Elk, 2015; Narmashiri, Sohrabi & Hatami, 2018). These orientations reflect learning through experiences in a particular culture (Van Elk, 2013).

There is a significant relationship between free will and paranormal beliefs (Mogi, 2013). Ogenler & Yapıcı (2012) concluded that more than half of the students had experienced paranormal beliefs at least once. One of the main reasons for the prevalence of paranormal beliefs is the lack of scientific knowledge about the nature of scientific processes (Mehmet & Ezgi, 2014). An increase in the popularity of the paranormal beliefs seems to be a threat to teaching science since it can cause competition and as a result, the public is less benefitted from the science (Mehmet & Ezgi, 2014). However, Stieger, Gumhalter, Tran, Voracek, and Swami (2013) showed a relationship between paranormal beliefs and conspiracy theories. Ilori, Adebayo, & Ogunleye (2014) found that paranormal beliefs have few effects on the tendency towards psychological trauma; however, religiosity does not affect psychological trauma. Moreover, Riekki, Lindeman, Aleneff, Halme, and Nuortimo, (2013) indicated a relationship between paranormal beliefs and perceiving the unreal pattern.

Coltheart (2007) suggested that the neural instability at the beginning of formation on the genome may affect the paranormal beliefs in the future. He also showed that the right hemisphere of the brain is responsible for the formation of illusion. Mohr et al., (2003) reported that the formation of these paranormal beliefs in both brain hemispheres could have dynamic and counter interactions. Tobacyk and Milford (1983) introduced three criteria to recognize three paranormal beliefs: 1. A phenomenon that cannot be explained by the present scientific knowledge; 2. A phenomenon that can be explained only by restricting and revising the laws of the science; and 3. A phenomenon that is incompatible with real normative deductions and expectations. Ramsey, Venette, and Rabalais (2011) identified a link between the tendency towards paranormal beliefs, i.e. the hidden, weird phenomena and interest towards conspiracy theory. Therefore, the personality and its relation to the paranormal beliefs can contribute to believing the conspiracy theory (Darwin, Neave& Holmes, 2011). The schizotypal disorder is highly associated with paranormal beliefs that affect the cognitive, perceptual, and emotional constructs (Meehl, 1990) and special religious beliefs are then involved by aging (Dreyer, Pieterse & Van, 1999). Narmashiri et al., (2018) studied on paranormal beliefs and delusion and suggested that information processing and reasoning orientations are the basis of the two thoughts.

Several studies have indicated that paranormal beliefs are accompanied by the right hemisphere processing (Narmashiri, Sohrabi & Hatami, 2017). Murphy and Lester (1976) showed that in a pious man, paranormal beliefs are essential in the right hemisphere processing. Brugger and Baumann (1994), Roig and Neaman (1992), and Thalbourne (1984) reported similar results. Electrophysiological findings have shown that the right hemisphere has a significant role in processing the paranormal beliefs Mohr et al., (2003). Leonard and Bruger (1998) showed that people with paranormal beliefs have low left hemisphere performance in their verbal activities. Due to the recent global studies on paranormal beliefs, it is of great importance to investigate this issue in Iran, as well. Thus, this research aimed at exploring the role of lateralization of the brain hemispheres and gender in paranormal beliefs.

## 2. Methods

The study population included all male and female students of the Sanandaj universities, Sanandaj City, Iran, ranged 18–29 years during the 2015–2016 academic year. Of them 180 students were selected by the convenience sampling method based on the handedness inventory as well as respecting all the principles of the university. They were informed about voluntary participation in the study. Because of the causal-comparative design of the study, as well as other relevant studies, 100 left-brained males, 6 left-brained females, 56 both left- and right-brained males and 22 both left-and right-brained females were selected as the final samples.

### 2.1. Study Instruments

#### 2.2.1. Paranormal Belief Scale (Blackmore & Moore, 1994)

This paranormal belief scale includes 10 statements about paranormal beliefs like “I have had at least one experience of having a relationship between my thoughts and those of another person.” The participants were asked to choose one of the options that fit them best. The lower scores indicate more beliefs towards paranormal issues. This scale has 10 questions plus 5 options ranged from completely agree to completely disagree. The lower scores on this scale show higher paranormal belief scores. The internal consistency coefficient for this scale was 0.87 implying its high coefficient.

#### 2.2.2. Velez Rudolf Wagner’s Brain Lateralization Scale (1985)

To measure the dominance of the brain hemispheres, we employed Velez Rudolf Wagner’s Brain Lateralization Scale (1985). To calculate the reliability of the scale of brain lateralization, we calculated the internal consistency coefficient or the Cronbach alpha coefficient. Accordingly, the internal consistency coefficient was calculated as 0.89, suggesting a high coefficient for the scale (Ahadi and Eslahkar, 2014).

## 3. Results

**Figure 1.**
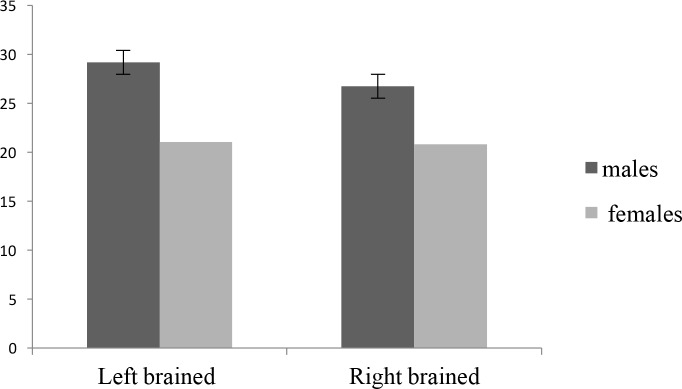
Comparing the means of paranormal scores in the studied groups

The obtained descriptive data are presented in Table 1 and Figure 1. The Mean±SD of the scores of paranormal beliefs in the male right-brained participants, female left-brained participants, both left- and right-brained males, and both left- and right-brained females were 29.16 (7.56), 21.00 (1.54), 26.71 (7.82), and 20.81 (5.16), respectively.

**Table 1.** The scores of paranormal beliefs for brain lateralization groups in males and females

**Variable**	**Groups**		**Min**	**Max**	**Mean**±**SD**
Paranormal beliefs	Left-brained	Males	13	47	29.16±7.56
		Females	19	22	21.00±1.54
	Right-brained	Males	10	45	26.71±7.82
		Females	12	33	20.81±5.16

To compare the effect of lateralization on the paranormal belief scores, univariate analysis of variance was employed for the scores of paranormal beliefs in the left-brained and both left- and right-brained participants. The findings showed that the obtained F statistic was significant (0.003) regarding the effect of lateralization on the scores of paranormal beliefs (9.338) with the degrees of freedom of 1 and 178. Therefore, there was a significant difference between the left-brained and both left- and right-brained participants in terms of the paranormal beliefs (Table 2).

**Table 2.** Comparison of the scores of paranormal beliefs in studied groups

**Source**	**Mean of Squares Total**	**Degrees of Freedom**	**Mean of Scores**	**F**	**Sig.**
Residuary	544.429a	1	544.429	9.338	0.003
Intercept	127737.051	1	127737.051	2190.872	0.001
Lateralization	544..429	1	544.429	9.338	0.003
Error	10378.149	178	85.304		
Total	145184.000	180			
Total Residuary	10922.578	179			

Also, to specify the demographic information (gender, education, and age) in the paranormal beliefs, linear regression analysis was utilized. The obtained F value from the linear regression analysis was significant (0.001) regarding the effect of brain lateralization on paranormal beliefs scores by gender (7.52). Thus, there was a significant difference between the scores of paranormal beliefs for the left-brained participants and both left- and right-brained participants (Table 3). However, no significant difference was observed in scores of paranormal beliefs in terms of education and age.

**Table 3.** Regression coefficients for prediction of the scores of paranormal beliefs based on the demographic information

**Variable**	**Predictor variables**	**Coefficient b**	**R**	**R^2^**	**F**	**Beta**	**T**	**Sig.**
Paranormal beliefs	Residue (a)	39.36	0.33	0.11	7.52	-----	11.08	0.001
	Gender	−7.06				−0.32	−4.41	0.001
	Education	−0.04				−0.006	−0.08	0.93
	Age	−0.16				−0.090	−1.25	0.21

## 4. Discussion

The most important finding of this study was a significant difference between the left-brained and both left-/right-brained participants in paranormal beliefs, and this difference was not observed between the left-brained females and both left- right-brained males as females achieved more paranormal beliefs.

Based on the obtained findings, brain hemispheres are lateralized in paranormal beliefs and its overlapping variable, i.e. conspiracy theory. This overlapping and the relationship between paranormal beliefs and conspiracy theory have been shown in many studies (Stieger et al., 2013). Also, based on previous studies, brain hemispheres play a pivotal role in paranormal beliefs. The results of this study indicate that the higher scores of the right brain hemisphere lead to more paranormal beliefs. Reviewing the available studies on the paranormal beliefs show that the right hemisphere is dominant and has been more considered in studies. Our findings are in line with those of Leonard and Brugger (1998), Pizagli et al., (2001), Mohr et al., (2003), Brugger and Baumann (1994), and Schulter and Papousek (2008).

Brugger and Baumann (1994) asserted that there was a probable relationship between the right hemisphere processing and perception and wrong belief. According to Bruger and Taylor (2003), the hyperactivity of the right hemisphere in comparison with the left hemisphere is specified by the fact that there is a correlation between the higher levels of beliefs towards the paranormal beliefs plus a deviation to the left side in the tactile rod division and implicit line division approaches. Brugger and Baumann (1994) claimed that the assumed relationship between the hyperactivity of the brain hemisphere and paranormal beliefs supports the findings in the studies on the divided-visual field in vocabulary processing. Brugger and Baumann (1994) reported that compared with the common pattern of left hemisphere dominance (e.g. right visual field) for the vocabulary processing in non-believers, those believing in paranormal phenomena more tend to have right-brain processing. Additionally, Pizzagalli et al., (2000) reported that the resting EEG pattern results in more right hemisphere processing in the believers. Pizzagalli et al., (2000) investigated the relationship between far-fetched motives and found that the ability of the believers to perceive the information is indirectly associated with the left visual field or the right hemisphere. Thus, based on the evidence, those who believe in paranormal phenomena may have a stronger tendency towards perceiving and recognizing a pattern in random motives. This can be due to the wrong concept of true random or a tendency towards establishing communications between different concepts.

As mentioned previously, females obtain a higher score in paranormal beliefs, which has been supported by other researchers (Schulter & Papousek, 2008); however, some researchers have found no difference between gender and paranormal beliefs (Fox & Williams, 2000). It seems that gender differences in paranormal beliefs are due to other factors, such as culture. Besides, a tendency towards paranormal beliefs cannot be due to the effect of education in a particular culture. Gender difference occurs during adolescence (Preece & Baxter, 2000) and since the identity is constructed through this period, we cannot overlook the role of tradition, religion, ethnic, abnormal culture-related beliefs, and particular religious and cultural training in gender differences (Thalbourne et al., 1995).

## 5. Conclusion

In general, the mentioned studies have illustrated that the brain hemispheres may have an essential role in paranormal beliefs. Also, personal differences play a significant role in paranormal beliefs. These results support the effects of learning and culture on the paranormal beliefs in other studies (Riekki, 2013; Lindeman & Svedholm 2012). Therefore, studying the socio-cultural effects on supernatural beliefs can be helpful.

## Ethical Considerations

### Compliance with ethical guidelines

All ethical principles were considered in this article. The participants were informed about the purpose of the research and its implementation stages; they were also assured about the confidentiality of their information; Moreover, They were allowed to leave the study whenever they wish, and if desired, the results of the research would be available to them.
